# Survivin protein expression is involved in the progression of non-small cell lung cancer in Asians: a meta-analysis

**DOI:** 10.1186/s12885-016-2304-3

**Published:** 2016-04-18

**Authors:** Liang Duan, Xuefei Hu, Yuxing Jin, Ruijun Liu, Qingjun You

**Affiliations:** Department of Thoracic Surgery, Shanghai Pulmonary Hospital, Tongji University School of Medicine, Shanghai, China; Department of Thoracic and Cardiovascular Surgery, Wuxi Fourth People’s Hospital (The Fourth Affiliated Hospital of SuZhou University), Wuxi, China

**Keywords:** Survivin, Non-small cell lung cancer, Pathological characteristics, Meta-Analysis

## Abstract

**Background:**

Surviving expression might serve as a prognostic biomarker predicting the clinical outcome of non-small cell lung cancer (NSCLC). The study was conducted to explore the potential correlation of survivin protein expression with NSCLC and its clinicopathologic characteristics.

**Methods:**

PubMed, Medline, Cochrane Library, CNKI and Wanfang database were searched through January 2016 with a set of inclusion and exclusion criteria. Data was extracted from these articles and all statistical analysis was conducted by using Stata 12.0.

**Results:**

A total of 28 literatures (14 studies in Chinese and 14 studies in English) were enrolled in this meta-analysis, including 3206 NSCLC patients and 816 normal controls. The result of meta-analysis demonstrated a significant difference of survivin positive expression between NSCLC patients and normal controls (RR = 7.16, 95 % CI = 4.63-11.07, *P* < 0.001). To investigate the relationship of survivin expression and clinicopathologic characteristics, we performed a meta-analysis in NSCLC patients. Our results indicates survivin expression was associated with histological differentiation, tumor-node-metastasis (TNM) stage and lymph node metastasis (LNM) (RR = 0.80, 95 % CI = 0.73-0.87, *P* < 0.001; RR = 0.75, 95 % CI = 0.67-0.84, *P* < 0.001; RR = 1.14, 95 % CI = 1.01-1.29, *P* = 0.035, respectively), but not pathological type and tumor size. (RR = 1.00, 95 % CI = 0.93-1.07, *P* = 0.983; RR = 0.95, 95 % CI = 0.86-1.05, *P* = 0.336, respectively).

**Conclusion:**

Higher expression of survivin in NSCLC patients was found when compared to normal controls. Survivin expression was associated with the clinicopathologic characteristics of NSCLC and may serves as an important biomarker for NSCLC progression.

## Background

Non-small cell lung cancer (NSCLC) remains one of the most fatal health problems in terms of morbidity and mortality and is the leading cause of cancer-related mortalities worldwide [1]. Histologically, NSCLC is consisted of three different subtypes: squamous cell carcinoma, adenocarcinoma, and large cell carcinoma, accounting for approximately 80 % ~ 85 % of lung cancer [2]. NSCLC is highly resistant to the existing cancer therapeutics and the great majority of NSCLC patients are diagnosed at advanced tumor stage. Although the recent advances in clinical and experimental oncology the survival of advanced NSCLC are still poor, with a 5-year survival rate of about 15 % [3, 4].

It is generally accepted that abnormal inhibition of apoptosis during homeostasis plays an important role in cancer development, progression and resistance to therapy [5]. Survivin, the common member of the inhibitor of the apoptosis protein (IAP) family, is a protein encoded by the BIRC5 gene in human with dual role in promoting cell proliferation and preventing apoptosis [6]. Previous studies revealed that survivin expression was found in precancerous lesions as well as in early stages of cancer in the skin, uterine cervix, colon, and oral mucosa [7, 8]. It was reported that survivin expression might serve as a prognostic biomarker predicting the clinical outcome of NSCLC, and might be associated with the clinicopathologic characteristics of NSCLC [5]. Perobska I et al. showed that lymph node metastases, tumor node metastasis (TNM) stage and tumor size had a higher incidence of survivin expression [9]. In order to clarify the relation between survivin expression and NSCLC, we conducted this meta-analysis.

## Methods

### Publication search

Online electronic databases (PubMed, Medline, Cochrane Library, CNKI and Wanfang) were searched with the key terms: (survivin or survivin protein) and (non-small cell lung cancer or NSCLC or non-small-cell lung carcinoma) (update to January 2016). We also checked out the reference lists of all retrieved studies and relevant reviews manually for important cross-references.

### Inclusion and exclusion criteria

Published studies were selected if they met all of the following criteria: (1) The study must be conducted in NSCLC patients; (2) The study must evaluate the Survivin protein expression; (3) Sufficient data, especially survivin positive expression in NSCLC patients and normal controls, have been provided to calculate risk ratios (RR) and 95 % confidence interval (95 % CI); (4) Number of NSCLC cases in enrolled studies should be more than 60; (5) The study must be published in a peer-reviewed journal; (6) The study must be independent from other studies. The exclusion criteria were as follows: (1) The studies did not conform to the inclusion criteria; (2) Reviews, case reports, editorials, guidelines and comments were excluded; (3) In case of duplicated publications or studies with overlapping data, the study with largest data was selected.

### Data extraction and qualitative assessment

The following data were collected from all the included studies: first author, publication year, country, ethnicity of participants, language, and numbers of participants, age, gender, subcellular localization and positive expression of survivin. Data from the finally selected studies were extracted based on a standard protocol. Potential discrepancy was resolved by discussions or by consulting the original report. Two reviewers independently assessed the methodological quality of the included trials using the Newcastle-Ottawa Scale (NOS) criteria to ensure consistency in reviewing and reporting results. The studies were scored based on three aspects: (1) selection of study group; (2) comparability of study groups; (3) ascertainment of the outcome of interest. A study was considered as low, moderate or high quality with the score 0 ~ 3, 4 ~ 6 and 7 ~ 9, respectively. Disagreement was settled by discussion, or a third investigator was consulted.

### Statistical analysis

Statistical test was conducted with the STATA statistical software (Version 12.0, Stata Corporation, College Station, TX, USA). To assess the correlation between survivin protein expression and the clinicopathologic characteristics, RR and its 95 % CI were calculated using random effects model or fixed-effects model. The statistical significance of pooled RRs was estimated by the application of Z test. We used Cochran’s Q-statistic (*P* < 0.05 was considered significant) and *I*^*2*^ test to assess heterogeneity among studies. Random effects model was applied for the evidence of significant heterogeneity (*P* < 0.05 or *I*^*2*^ test exhibited > 50 %); otherwise, fixed-effects model was used. Univariate and multivariate meta-regression analyses were used to evaluate the potential sources of heterogeneity. Further identification was performed by using Monte Carlo method. Additionally, we applied a sensitivity analysis to evaluate whether one single study had the weight to impact on the overall estimate. Further, the effect of publication bias was examined by Egger’s linear regression test (*P* < 0.05 was considered significant).

## Results

### Literature searching results and baseline characteristics of included studies

**Fig. 1:**
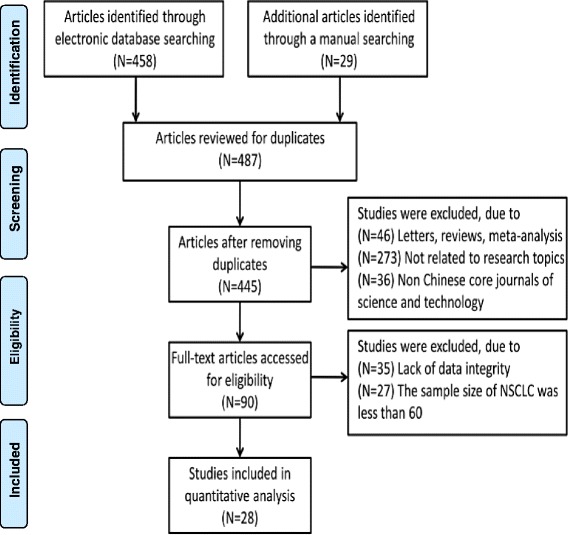
PRISMA Flow chart of study selection procedure

Four hundred and eighty-seven articles were initially identified through database searches. Twenty-eight studies remained after excluding duplicates (*n* = 42), letters, reviews, meta-analyses (*n* = 46) and irrelevant topic (*n* = 273), non-core journal in Chinese (*n* = 36), insufficient information in studies (*n* = 35) and number of NSCLC cases less than 60 (*n* = 27), 28 trials were finally selected for this meta-analysis (Fig. 1) [5, 10–36]. The enrolled studies published between 2005 and 2015 included 3206 NSCLC patients and 816 normal controls, with 2252 males and 954 females. For the pathological type, 1010 patients with squamous cell carcinoma (SCC), 806 with adenocarcinoma (AC). With respect to clinicopathologic features, 1198 patients with well/moderate differentiation, 788 with poor differentiation; 1421 at I/II stage and 1009 at III/IV stage (TNM stage); 1256 patients with lymphatic metastasis and 1185 patients without lymphatic metastasis. All included studies scored 7 in terms of NOS scores. The baseline characteristics of included studies were showed in Table 1.

**Table 1:** Baseline characteristics of included studies

First author	Year	Country	Ethnicity	Language	Disease	Method	Case Number	Sample source	Gender (M/F)	Age (years)
Hirano H	2015	Japan	Asians	English	NSCLC	IHC	157	tissue	115/42	66.7(47–82)
Hu S	2013	China	Asians	English	NSCLC	IHC	256	tissue	176/80	57.7
Sun PL	2013	Korea	Asians	English	NSCLC	IHC	373	tissue	258/115	65.0(21–84)
Zhang XY	2012	China	Asians	Chinese	NSCLC	IHC(SP)	60	tissue	35/25	54.0(30–78)
Peng X	2012	China	Asians	English	NSCLC	IHC	97	tissue	75/22	58.3(28–75)
Wang M	2012	China	Asians	English	NSCLC	IHC	210	tissue	130/80	59.8(35–76)
Gao Q	2012	China	Asians	English	NSCLC	IHC	62	tissue	44/18	57.8(35–78)
Hu FQ	2011	China	Asians	Chinese	NSCLC	IHC(Envision)	116	tissue	78/38	65.8(35–84)
Guosheng L	2011	China	Asians	English	NSCLC	IHC(SP)	100	tissue	69/31	55.6(37–76)
Fan CF	2011	China	Asians	English	NSCLC	IHC	76	tissue	46/30	57.1(26–78)
Zhu CZ	2010	China	Asians	Chinese	NSCLC	IHC(SP)	60	tissue	39/21	62.1(33–78)
Yang DX	2010	China	Asians	Chinese	NSCLC	IHC(PowerVision)	60	tissue	40/20	53.5(37–71)
Zeng ZH	2010	China	Asians	Chinese	NSCLC	IHC	60	tissue	38/22	65.7(40–78)
Porebska I	2010	Poland	Caucasians	English	NSCLC	IHC	74	tissue	49/25	60.5(43–77)
Chen YQ	2009	China	Asians	English	NSCLC	IHC(SP)	120	tissue	94/26	61.0(42–76)
Li CH	2008	China	Asians	Chinese	NSCLC	IHC(PV)	91	tissue	77/14	62.0(39–78)
Shi M	2007	China	Asians	Chinese	NSCLC	IHC	80	tissue	55/25	56.2(33–79)
Miao LJ	2007	China	Asians	Chinese	NSCLC	IHC(SP)	80	tissue	53/27	58.8(18–78)
Xue ZX	2006	China	Asians	Chinese	NSCLC	IHC(SP)	84	tissue	51/33	53.2(22–75)
Wang M	2006	China	Asians	Chinese	NSCLC	IHC	72	tissue	45/27	58.5(38–74)
Li XC	2006	China	Asians	Chinese	NSCLC	IHC(SABC)	64	tissue	41/23	55.6(35–78)
Yoo J	2006	Korea	Asians	English	NSCLC	IHC	219	tissue	168/51	65.8 ± 9.9
Huo XD	2006	China	Asians	Chinese	NSCLC	IHC(Envision)	117	tissue	85/32	57.5(29–71)
Vischioni B	2006	Netherlands	Caucasians	English	NSCLC	IHC	160	tissue	129/31	64.0(40–86)
Akyurek N	2006	Turkey	Caucasians	English	NSCLC	IHC	78	tissue	72/6	60.8(39–78)
Ren YJ	2006	China	Asians	Chinese	NSCLC	IHC(Envision)	61	tissue	45/16	62.0(40–75)
Qiu HL	2005	China	Asians	Chinese	NSCLC	IHC(SP)	75	tissue	51/24	57.1 ± 10.6
Shinohara ET	2005	America	Caucasians	English	NSCLC	IHC	144	tissue	94/50	65.4 ± 11.04

(Notes: NSCLC = non-small cell lung cancer; IHC = Immunohistochemical;M = male; F = female; OA = osteoarthritis)

### The comparison between NSCLC patients and normal controls on survivin protein expression

**Fig. 2:**
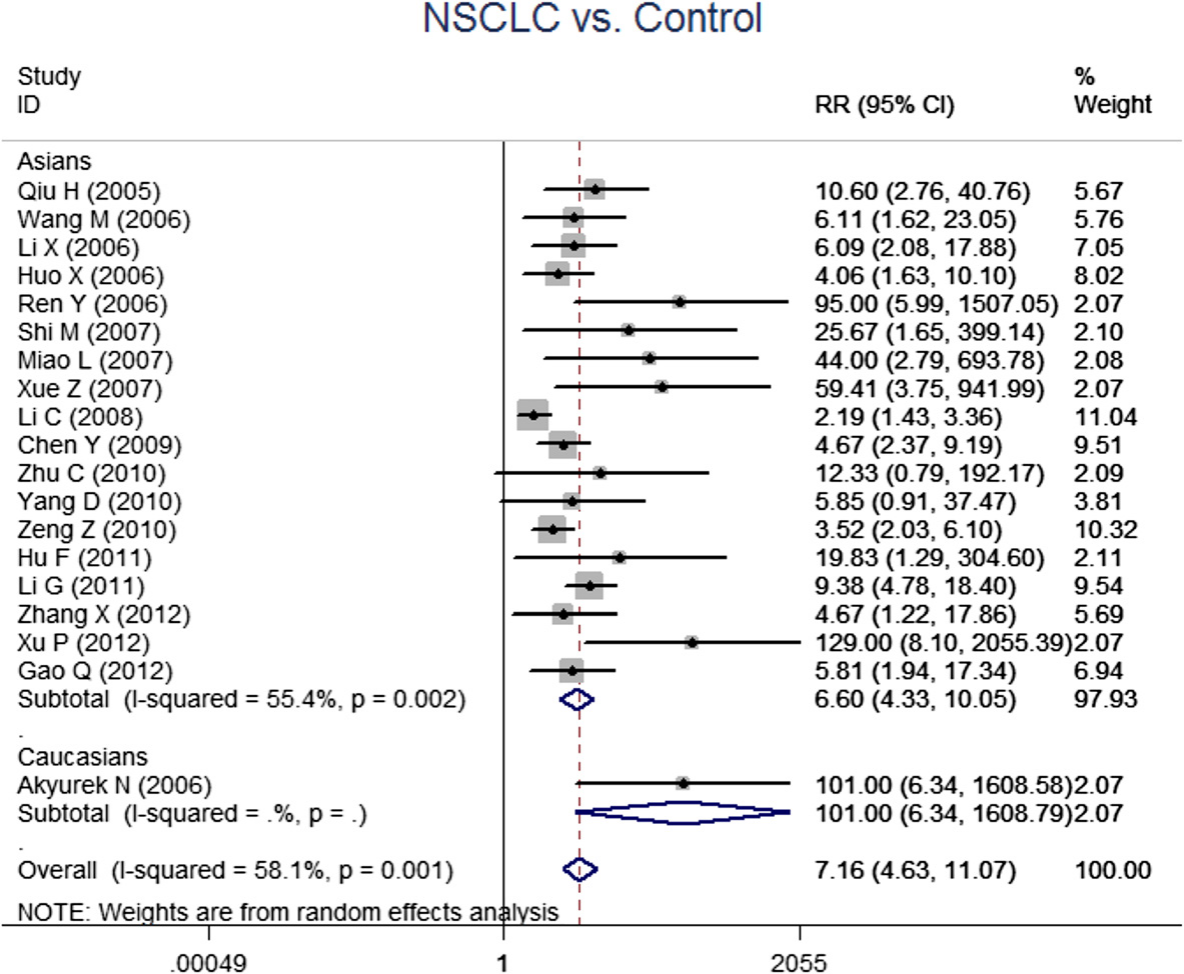
Forest plots for the comparisons of survivin expression between NSCLC patients and normal controls

A total of 19 studies provided data of survivin expression in NSCLC patients and normal controls (1537 NSCLC patients and 816 normal controls). Heterogeneity test revealed the existence of heterogeneity in those 19 trials, thus a random-effect model was used (*I*^*2*^ = 58.1 %, *P* < 0.001). Meta-analysis result revealed that survivin expression in NSCLC patients was significantly higher when compared with normal controls (RR = 7.16, 95 % CI = 4.63-11.07, *P* < 0.001) (Fig. 2).

### The analysis of survivin expression and clinicopathologic characteristics of NSCLC

**Fig. 3:**
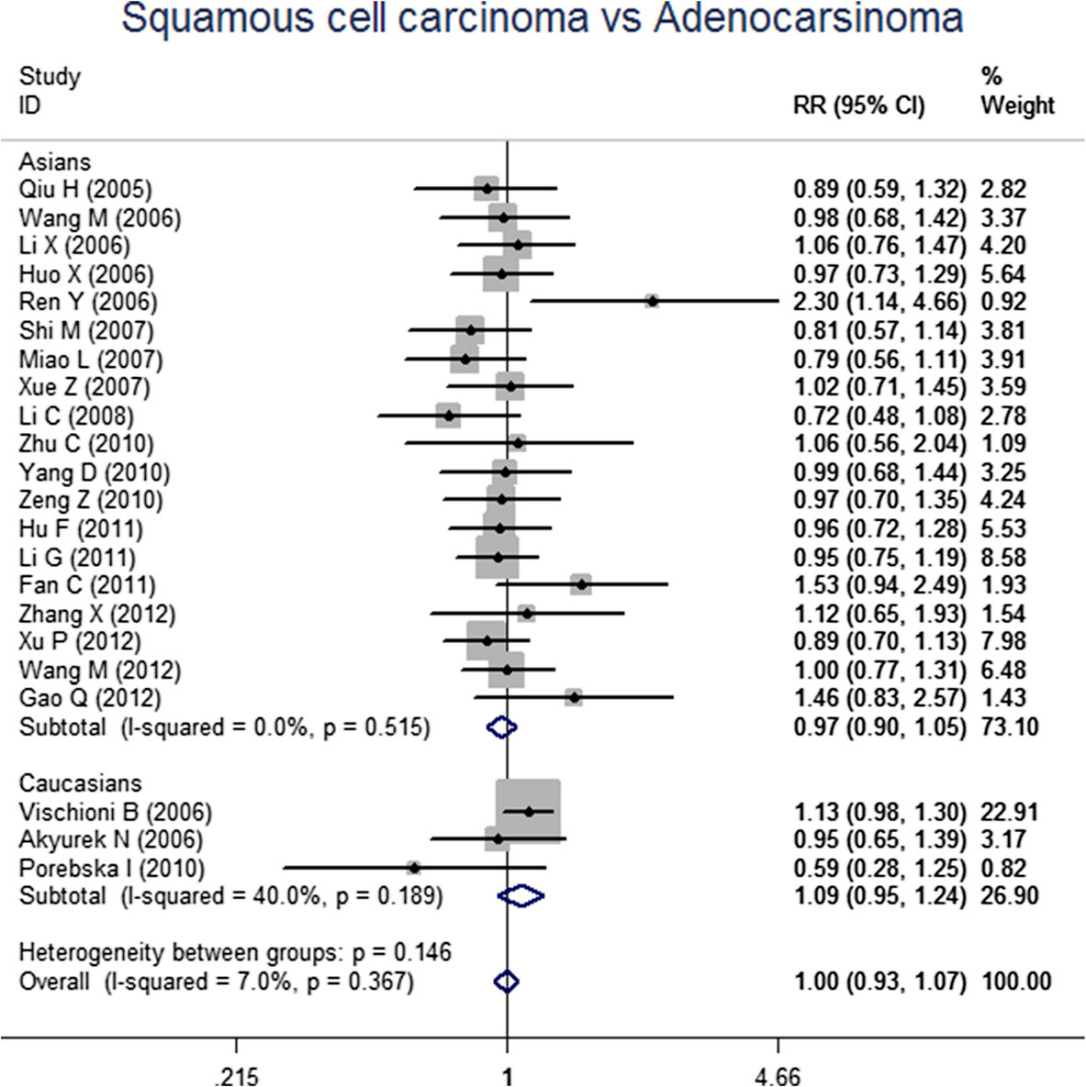
Forest plots for the comparisons of survivin expression between SCC patients and AC patients

**Fig. 4:**
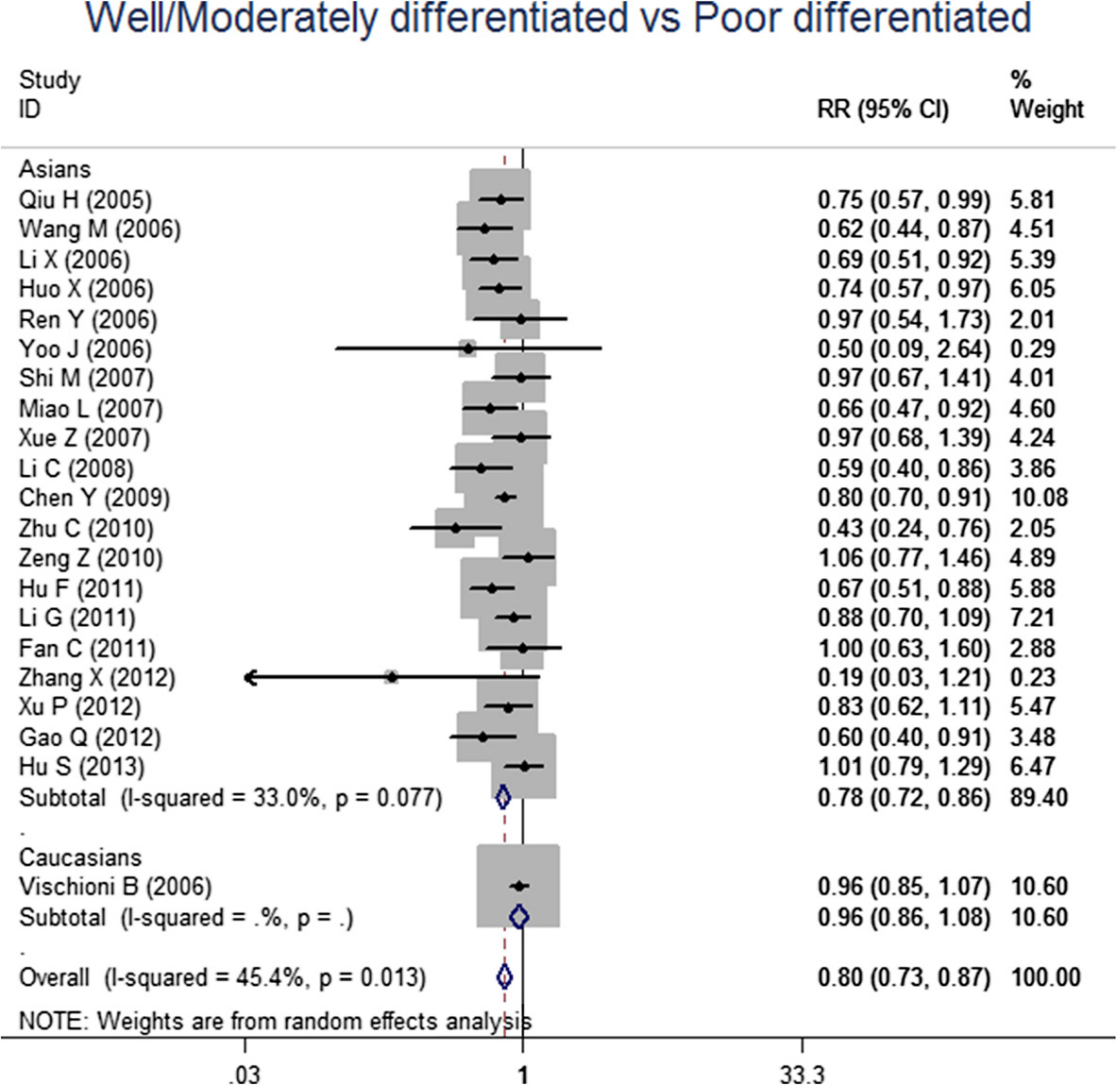
Forest plots for the comparisons of survivin expression between well/moderated differentiated patients and poor differentiated patient

**Fig. 5:**
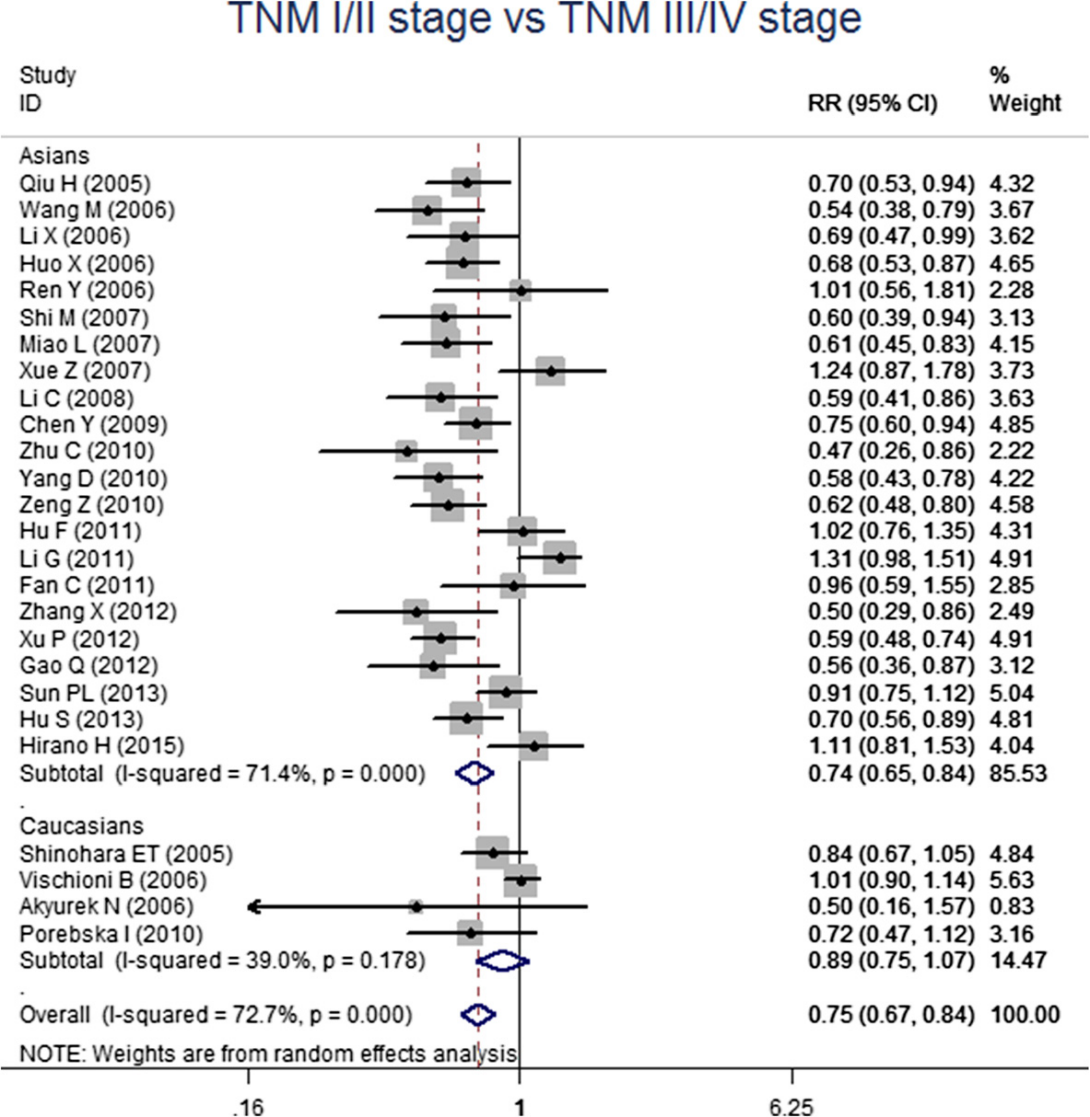
Forest plots for the comparisons of survivin expression between patients at TNM I/II stage and TNM III/IV stage

**Fig. 6:**
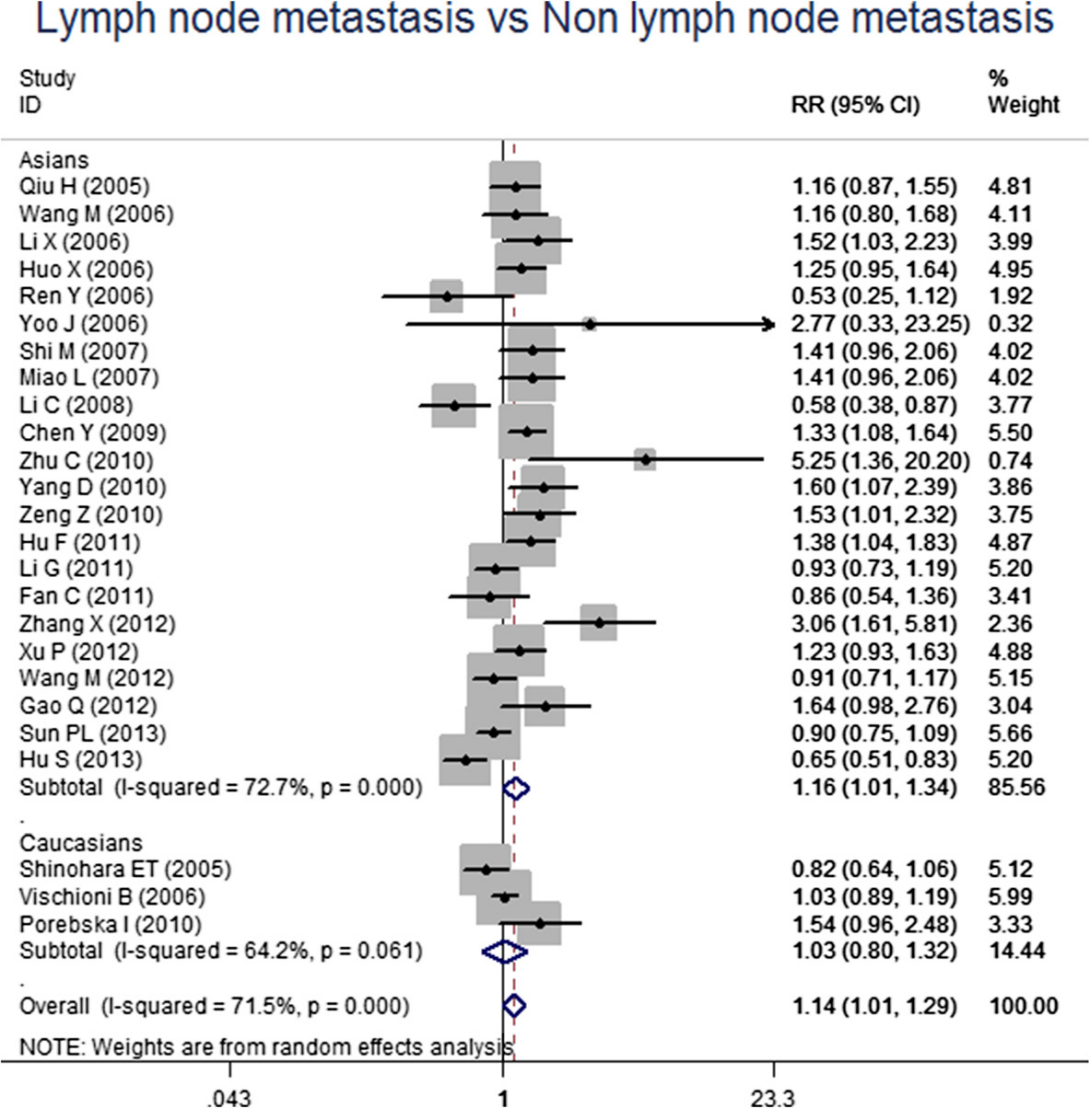
Forest plots for the comparisons of survivin expression between patients with LNM and without LNM

**Fig. 7:**
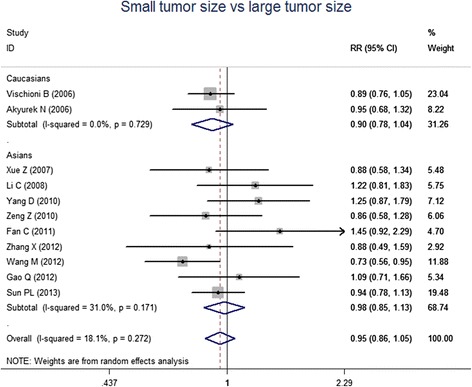
Forest plots for the correlation of survivin expression and tumor size

For the meta-analysis according to pathological types, we included 22 studies, involving 1010 SCC patients and 806 AC patients. Heterogeneity test revealed the lack of heterogeneity in these studies and a fixed-effect model was applied (*I*^*2*^ = 7 %, *P* = 0.367). No significantly different survivin expression was found between squamous cell carcinoma (SCC) and adenocarcinoma (AC) (RR = 1.00, 95 % CI = 0.93-1.07, *P* = 0.983) (Fig. 3). A total of 21 studies investigated histological differentiation of NSCLC patients and moderate heterogeneity existed in these studies (*I*^*2*^ = 45.4 %, *P* = 0.013). Results from random-effect model suggested that survivin expression was significantly lower in NSCLC patients with well/moderate differentiation than that in the patients with poor differentiation (RR = 0.80, 95 % CI = 0.73-0.87, *P* < 0.001) (Fig. 4). 26 studies provided survivin expression level at different TNM stages. Heterogeneity test showed the presence of heterogeneity in these studies (*I*^*2*^ = 72.7 %, *P* < 0.001). Meta-analysis results revealed that NSCLC patients at TNM III/IV stage had a significantly higher survivin expression than the patients at TNM I/II stage (RR = 0.75, 95 % CI = 0.67-0.84, *P* < 0.001) (Fig. 5). A total of 25 studies indicated the status of lymphatic metastasis. Meta-analysis suggested that survivin expression in NSCLC patients with lymphatic metastasis was significantly higher than that in the patients without lymphatic metastasis (RR = 1.14, 95 % CI = 1.01-1.29, *P* = 0.035) (Fig. 6). 11 studies showed the survivin expression in the patient with different tumor size. No heterogeneity was found in these studies (*I*^*2*^ = 18.1 %, *P* = 0.272). Meta-analysis revealed that survivin expression was not associated with tumor size (RR = 0.95, 95 % CI = 0.86-1.05, *P* = 0.336) (Fig. 7).

We also performed subgroup analysis according to the ethnicity. And the results showed survivin expression was associated with respect to histological differentiation, TNM stage and lymph node metastasis in Asian populations but not in Caucasian populations. (Table 2) For Caucasians, only the contrast of NSCLC versus and normal control reach the statistical significance. According to the definition of positive expression, the studies were divided in to 3 subgroups. (1 Survivin expressed in cytoplasm only, 2 Survivin expressed in cytoplasm or nucleus, 3 Survivin expressed in both cytoplasm and nucleus) Subgroup analysis found survivin expression was associated with histological differentiation, TNM stage and lymph node metastasis in subgroup 1 and subgroup 2, but not in subgroup 3. (Table 3)

**Table 2:** Summary of subgroup analysis by ethnicity

Studies	Ethnicity (n)	Studies (n)	Combined RR (95 % CI)	P(Z)	I2	P(Q)
NSCLC vs. Control	All	19	7.16(4.63-11.07)	<0.001	58.1 %	0.001
	Asians	18	6.60(4.33-10.05)	<0.001	55..4 %	0.002
	Caucasians	1	101(6.34-1608)	0.001	/	/
Squamous cell carcinoma vs. Adenocarcinoma	All	22	1.00(0.93, 1.07)	0.983	7.0 %	0.367
	Asians	19	0.97(0.90-1.05)	0.44	0 %	0.189
	Caucasians	3	1.01(0.78-1.30)	0.959	40.0 %	0.515
Well/Moderately differentiated vs. Poor differentiated	All	21	0.80(0.73-0.87)	<0.001	45.4 %	0.013
	Asians	20	0.78(0.72-0.86)	<0.001	33 %	0.077
	Caucasians	1	0.96(0.86-1.08)	0.487	/	/
TNMI/II stage vs. TNM III/IVstage	All	26	0.75(0.67-0.84)	<0.001	72.7 %	<0.001
	Asians	22	0.74(0.65-0.84)	<0.001	71.4 %	<0.001
	Caucasians	4	0.89(0.75-1.07)	0.222	39 %	0.178
Lymph node metastasis vs. Non lymph node metastasis	All	25	1.14(1.01-1.29)	0.035	71.5 %	<0.001
	Asians	22	1.16(1.01-1.34)	0.037	72.7 %	<0.001
	Caucasians	3	1.03(0.80-1.32)	0.839	64.2 %	0.061
Small Tumor vs. Big Tumor	All	11	0.95(0.86-1.05)	0.336	18.1 %	0.272
	Asians	9	0.98(0.85-1.13)	0.796	31 %	0.171
	Caucasians	2	0.90(0.78-1.04)	0.161	0 %	0.729

**Table 3:** Summary of subgroup analysis by localization of survivin expression

Contrasts	Subcellular locolization	Study (n)	Combined RR (95 % CI)	P(Z)	I2	P(Q)
NSCLC vs. Control	All	19	7.16(4.63-11.07)	<0.001	58.1 %	0.001
	Cytoplasma	9	7.14(4.19-12.16)	<0.001	43.8 %	0.076
	Cytoplasma or nuclearus	5	3.96(1.93-8.14)	<0.001	46.1 %	0.115
	Cytoplasma and nuclearus	2	17.41(1.2-252.4)	0.036	70.5 %	0.065
Squamous cell carcinoma vs. Adenocarcinoma	All	22	0.99(0.92, 1.07)	0.866	7.0 %	0.367
	Cytoplasma	11	0.98(0.88-1.08)	0.641	0 %	0.767
	Cytoplasma or nuclearus	6	0.98(0.83-1.15)	0.771	30.8 %	0.204
	Cytoplasma and nuclearus	2	1.74(1.12-2.71)	0.013	0 %	0.324
Well/Moderately differentiated vs. Poor differentiated	All	21	0.80(0.73-0.87)	<0.001	45.4 %	0.013
	Cytoplasma	8	0.84(0.75-0.93)	0.001	19.6 %	0.274
	Cytoplasma or nuclearus	7	0.68(0.52-0.88)	0.003	68.9 %	0.004
	Cytoplasma and nuclearus	2	0.73(0.46-1.16)	0.179	42.7 %	0.186
TNMI/II stage vs. TNM III/IVstage	All	26	0.75(0.67-0.84)	<0.001	72.7 %	<0.001
	Cytoplasma	12	0.83(0.69-1.01)	0.059	74.1 %	<0.001
	Cytoplasma or nuclearus	8	0.73(0.61-0.88)	0.001	74.5 %	<0.001
	Cytoplasma and nuclearus	2	0.73(0.41-1.29)	0.278	59.7 %	0.115
Lymph node metastasis vs. Non lymph node metastasis	All	25	1.14(1.01-1.29)	0.035	71.5 %	<0.001
	Cytoplasma	10	1.24(1.07-1.44)	0.005	53.1 %	0.024
	Cytoplasma or nuclearus	9	1.12(0.89-1.41)	0.352	76.6 %	<0.001
	Cytoplasma and nuclearus	2	0.96(0.32-2.91)	0.949	83.1 %	0.015
Small Tumor vs. Big Tumor	All	11	0.95(0.86-1.05)	0.336	18.1 %	0.272
	Cytoplasma	6	0.97(0.79-1.19)	0.738	48.3 %	0.085
	Cytoplasma or nuclearus	4	0.92(0.83-1.04)	0.226	0 %	0.565
	Cytoplasma and nuclearus	1	1.09(0.71-1.67)	0.691	/	/

### Sensitivity analysis and publication bias

The sensitivity analysis demonstrated that a single study had no significant effect on the pooled RRs. Egger’s test based on the 19 literatures which provided the comparison between NSCLC patients and normal controls revealed the presence of publication bias (*P* = 0.001). After the application of fill and trim method, statistical significance still existed on the survivin expression between NSCLC patients and normal controls (*P* < 0.001), suggesting publication bias has no significant effect on the final results. For those studies investigated pathological types (*n* = 22), histological differentiation (*n* = 21), TNM stage (*n* = 26), lymphatic metastasis (*n* = 25) and tumor size (*n* = 11), no publication biases were found by Egger’s test.

### Meta-regression analysis

Univariate meta-regression analysis revealed that country and ethnicity may be the potential sources for most of heterogeneity (*P* > 0.05). Multivariate meta-regression analysis further confirmed this finding (Table 4).

**Table 4:** Meta-regression analyseis of potential source of heterogeneity

Heterogeneity factors	Coefficient	SE	t	*P*	95 % CI	
					LL	UL
Country	82.25	25.89	3.18	0.037	27.37	137.13
Ethnicity	78.35	27.13	3.08	0.025	26.15	120.65
Language	8.82	18.07	0.49	0.598	−29.49	47.12
Sample Size	−0.18	0.37	−0.49	0.234	−26.96	102.39

(Notes: SE = Standard Error; LL = Lower Limit; UL = Upper Limit)

## Discussion

The tumorigenesis of NSCLC is a complex process with the feature of imbalance in cell apoptosis and proliferation. Aberrant proliferation of tumor cells may emerge as cell apoptosis is inhibited, which eventually provided supports for tumorigenesis, development, invasion and metastasis [37]. Survivin is one of the most important inhibitor of IAP family, which is normally expressed in embryonic and fetal tissues but is almost absent in terminally differentiated cells [6, 38]. Its overexpression has been reported in many malignancies including NSCLC. [39] Several studies have reported survivin overexpression was involved in the development of NSCLC [7, 8].

The result of meta-analysis showed a significant difference in survivin expression between NSCLC patients and normal controls. To investigate the correlation between survivin expression and clinicopathologic characteristics, we performed several meta-analysis in NSCLC patients classified by clinicopathologic parameters. Our results suggested survivin expression was associated to histological differentiation, tumor-node-metastasis (TNM) stage and lymph node metastasis (LNM). Roles of survivin in the progression of NSCLC have been investigated previously. Babaei et al. reported survivin is associated with high grade malignancies. [40] Significant overexpression of survivin was observed in NSCLC patients at late stage. [41] A strong heterogeneity was detected among individual studies. Meta-regression indicated ethnicity was the primary source of heterogeneity. In the subgroup analysis classified by ethnicity, the significant associations were still present in Asians but not in Caucasians. One possible reason was that only few studies were conducted in Caucasians and no firm conclusions can be draw from a small sample set. Further research with large sample size is needed to define the impact of survivin expression in Caucasians.

Survivin has been shown to localize in mitochondria, cytoplasm and nucleus. And the functional dynamics of survivin are dependent on its subcellular localization. [42] Localization of survivin to the nucleus and cytoplasm confers its role in mitosis regulation and apoptosis inhibition. [43] In nucleus, survivin is involved in the chromosomal packaging complex and controls mitosis in many aspects including regulations of the mitotic spindle checkpoint and mitotic progression. [44] As an inhibitor in IAP family, survivin can directly inhibit caspase-3 and caspase-7 activity to prevent apoptosis [5]. In the studies included in our meta-analysis, most studies reported cytosol survivin expression only. Several studies defined positive expression as survivin expression in cytoplasm or nucleus. Only in 2 studies survivin expression in both cytoplasm and nucleus was considered as positive expression. We performed subgroup analysis according to the subcellular localization of survivin and only found the 2 studies with survivin expression in both cytoplasm and nucleus gave different results with other subgroups. Further research is necessary to determine with precision whether there is a correlation between subcellular localization of survivin expression and progression of NSCLC.

There were several limitations in our present meta-analysis. First, for the insufficiency of data, we did not analyze whether survivin expression is correlated the prognosis of NSCLC. Secondly, although our meta-analysis included 28 studies, only 4 studies were performed in Caucasians. Thus, no firm conclusions can be draw in Caucasians and the difference between Asians and Caucasians is uncertain.

## Conclusions

In conclusion, although our meta-analysis has some shortcomings, it still provides evidence that survivin expression was associated with the clinicopathologic characteristics of NSCLC in Asians, suggesting that survivin protein can serves as an important biomarker for the progression of NSCLC. However, further investigations with more integral data are needed to determine the correlation of survivin expression and the progression of NSCLC in Caucasians.

## Abbreviations

NSCLC: Non-small cell lung cancer
TNM: Tumor-node-metastasis
LNM: Lymph node metastasis
NOS: Newcastle-Ottawa scale
RR: Risk ratios
CI: Confidence interval
SCC: Squamous cell carcinoma
AC: Adenocarcinoma
IAP: Inhibitor of the apoptosis protein
